# *N*‐Doping Donor‐Dilute Semitransparent Organic Solar Cells to Weaken Donor: Acceptor Miscibility and Consolidate Donor‐Phase Continuity

**DOI:** 10.1002/advs.202404135

**Published:** 2024-06-17

**Authors:** Jiaqi Xie, Weihua Lin, Kaibo Zheng, Ziqi Liang

**Affiliations:** ^1^ Department of Materials Science Fudan University Shanghai 200433 China; ^2^ Department of Chemical Physics and NanoLund Lund University Box 124 Lund 22100 Sweden; ^3^ Department of Chemistry Technical University of Denmark Kongens Lyngby DK‐2800 Denmark; ^4^ Institute of Optoelectronics Fudan University Shanghai 200433 China

**Keywords:** donor dilute, hole transport, n‐doping, phase continuity and miscibility, semitransparent organic solar cells

## Abstract

Lightweight and semi‐transparent organic solar cells (ST‐OSCs) offer bright promise for applications such as building integrated photovoltaics. Diluting donor content in bulk‐heterojunction active layers to allow greater visible light transmittance (AVT) effectively enhances device transparency, yet the ineluctable compromise of the donor‐phase continuity is challenging for efficient charge transport. Herein, a trace amount of *n*‐type *N*‐DMBI dopant is incorporated, which facilitates the donor:acceptor (D:A) de‐mixing by strengthening both acceptor polarity and D/A crystallization. With the diminution of component inter‐mixing, the limited number of donors increasingly self‐aggregate to establish the more continuous phases. For the benchmark PM6:Y6‐based ST‐OSCs, when the donor content is reduced from regular 45 to optimal 30 wt.%, the device AVT is remarkably raised by more than a quarter, accompanied by a marginal drop in power conversion efficiency from 13.89% to 13.03%. This study reveals that by decreasing the donor content to <30 wt%, acceptor excitons induced by Förster resonance energy transfer are prone to severe radiative recombination. This is nonetheless mitigated by dopant inclusion within the acceptor phase by providing extra energy offset and prolonging charge transfer state lifetime to assist exciton dissociation.

## Graphical abstract

Diluting donor content can effectively enhance device visible transparency, yet the less continuous donor‐phase is troublesome for efficient charge transport. Incorporating *n*‐type dopants can strengthen acceptor polarity, as well as donor and acceptor (D/A) crystallizations, thereby delicately reducing D:A miscibility. Consequently, donor molecules are promoted to self‐aggregate to form more interconnective domains rather than intermixing with acceptors.

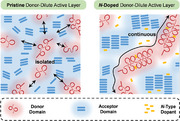

## 1 Introduction

Non‐fullerene organic solar cells (NF‐OSCs) have witnessed a remarkable breakthrough in power conversion efficiency (PCE) that is on the verge of 20%,^[^ ^1^ ^]^ evoking more enthusiasm for the application‐oriented research. Taking advantage of the exclusive merits of high transparency and colorful aesthetics, OSCs are particularly competitive on (semi‐)transparent devices such as building integrated photovoltaics. From 2006 to 2015, extensive efforts in the research of semi‐transparent organic solar cells (ST‐OSCs) have been devoted to exploiting transparent top electrodes such as ultra‐thin metals, silver nanowire composites, conductive polymers, etc.^[^ ^2^ ^]^ In spite of a relatively low average visible light transmittance (AVT) below 30%,^[^ ^3^ ^]^ the thermally evaporated Ag thin layer of 10–25 nm thickness is still widely employed as the top electrode to date, owing to the stringent necessities for energy level alignment, high electrical conductivity, and non‐destructive processing techniques. Thereafter, recent advancements in near‐infrared non‐fullerene acceptors (NFAs) and device engineering have propelled ST‐OSCs to acquire an AVT above the threshold of 20% and meanwhile maintain a reasonable PCE exceeding 13%.^[^ ^4^, ^5^ ^]^ For instance, the Zhu group systematically investigated the optimal optical spectral response up to 1100 nm for NFAs in high‐performance ST‐OSCs.^[^ ^6^ ^]^ Building on the derived theoretical foundation, they proposed a series of effective strategies to finely tune the acceptor bandgaps, which included enhancing quinoidal resonance,^[^ ^6^, ^7^ ^]^ adopting N‐substituted asymmetric structures,^[^ ^8^ ^]^ and fluorinating the core units.^[^ ^9^ ^]^

In principle, the active layer of OSCs adopts a bulk‐heterojunction (BHJ) structure casted from donor (D) and acceptor (A) blends, wherein the numerous D/A interfaces provide energy offsets to drive exciton dissociation while the relatively pure D/A phases establish a bi‐continuous network to afford the hole/electron transportation. To achieve efficient exciton dissociation and charge transport, the optimally sized phase‐separation in BHJ blends is preferred, which always requires a precise control of D/A mass ratio.

In high‐performance NF‐OSCs, the optical absorption of donor (300–700 nm) and acceptor (600–1000 nm) are well complementary, converting both visible and near‐infrared lights. Therefore, diluting the donor content can straightforward enhance the device transparency in the visible region. However, besides the ineluctable penalty in energy utilization, it will also reduce D/A interfaces and disconnect donor phase, which is detrimental to exciton dissociation and charge transport. Thanks to the special A−DA'D−A chemical structure of Y‐series NFAs,^[^ ^10^ ^]^ the intramolecular push‐pull effect^[^ ^11^ ^]^ and the dimerized intermolecular packing^[^ ^12^, ^13^, ^14^ ^]^ significantly lower the binding energy of excitons generated in the aggregates of acceptors, which even make the spontaneous exciton dissociation possible with an aid of the ambient thermal energy^[^ ^14^, ^15^ ^]^ and/or the energetic variation among crystalline and amorphous regions.^[^ ^16^, ^17^ ^]^ This superiority mitigates the dependence of exciton dissociation at D/A interfaces, but the issue of the less continuous donor phase remains challenging for the donor‐dilute ST‐OSCs.^[^ ^10^, ^18^ ^]^

Hole transport still seemed viable with the constitution of only 10 wt.% donor in high‐efficiency NF‐OSCs,^[^ ^19^, ^20^ ^]^ yet the extended inter‐domain distance and the lower donor concentration within the intermixed regions would reduce the probabilities of charge hopping^[^ ^20^ ^]^ and tunneling.^[^ ^21^, ^22^ ^]^ Some research works also suggested that the ambipolar nature of NFAs may support the transportation of electrons and holes. However, the inoperative single‐component (i.e., NFAs‐only) devices demonstrated that the continuous donor phase is necessary for effective hole transport by spatially isolating the separated electrons and holes, which will otherwise rapidly recombine.^[^ ^10^, ^23^ ^]^ Given the high D:A miscibility for most high‐performance norfullerene systems, diluting donor content in active layers would conceivably result in small sized and less interconnective donor domains, because both donor nuclei and domain critical size would get reduced to maintain thermodynamic and mass‐diffusion kinetic equilibria.^[^ ^24^, ^25^ ^]^

In order to reinforce the donor phase continuity in donor‐diluted active layers, hereby we propose to utilize *n*‐type doping to delicately reduce the D:A miscibility such that more donor materials can aggregate into relatively pure domains rather than intermix with acceptors. Our previous works found that *n*‐type doping can enhance the polarity of NFAs in the way of forming charge transfer complex with dopants.^[^ ^26^, ^27^ ^]^ Meanwhile, the inclusion of high surface‐tension dopants also can provide nucleation sites to lower the energetic barrier for solute precipitation, as well as promote the epitaxial growth of crystallites.^[^ ^26^, ^27^ ^]^ Therefore, such dual functionality of *n*‐type doping supports the concept of regulating the demixing of donor and acceptor components in BHJ active layers.

Here we still used the benchmark n‐type dopant, 4‐(1,3‐dimethyl‐2,3‐dihydro‐1H‐benzoimidazol‐2‐yl)phenyl) dimethylamine (i.e., *N*‐DMBI), to dope the representative NFAs of 2,2′‐((2Z,2′Z)‐((12,13‐bis(2‐ethylhexyl)−3,9‐diundecyl‐12,13‐dihydro‐[1,2,5]thiadiazolo[3,4‐e]thieno[2′,3′:4′,5′]thieno[2′,3′:4,5]pyrrolo[3,2‐g]thieno[2′,3′:4,5]thieno[3,2‐b]indole‐2,10‐diyl)bis(methanylylidene))bis(5,6‐difluoro‐3‐oxo‐2,3‐dihydro‐1H‐indene‐2,1‐diylidene))dimalononitrile) (i.e., Y6) to mediate its miscibility with the donor of poly[[4,8‐bis[5‐(2‐ethylhexyl)−4‐fluoro‐2‐thienyl]benzo[1,2‐b:4,5‐b′]dithiophene‐2,6‐diyl]−2,5‐thiophenediyl[5,7‐bis(2‐ethylhexyl)−4,8‐dioxo‐4H,8H‐benzo[1,2c:4,5c′]dithiophene‐1,3‐diyl]−2,5‐thiophenediyl] (i.e., PM6). Doping reaction between *N*‐DMBI and Y6 has been extensively validated in literature,^[^ ^28^ ^−^ ^30^ ^]^ in which the thermally‐activated C–H bond cleavage of *N*‐DMBI–H produces the intermediate [Y6–H^•^+*N*‐DMBI^•^] radicals and further ionized into [Y6^•–^+*N*‐DMBI^+^] via hydride transfer.^[^ ^31^, ^32^ ^]^

By utilizing a series of morphological characterizations and phase‐field simulation, we find that incorporating a small number of *N*‐DMBI dopants can effectively enlarge the donor domains by slightly reducing the miscibility between PM6 and Y6 in the active layer comprising only 30 wt.% PM6. Consequently, such donor‐dilute active layers acquire film structures similar to those comprising standard D:A compositions (that is, 1:1.2 by weight, ≈45 wt.% PM6), which substantially raised the AVT of the corresponding ST‐OSCs by more than a quarter whereas the PCE slightly dropped from 13.89% to 13.03%. Impressively, our investigation reveals that the photo‐generated excitons in donors are dissociated via directly injecting electrons to acceptors (i.e., electron transfer route) or undergoing Förster resonance energy transfer (FRET) followed by the hole injection from acceptors to donors (i.e., FRET route). Surprisingly, the FRET route seems to be less efficient than what has been anticipated in the absence of sufficient D/A interfaces.^[^ ^33^ ^]^ This is evidenced by the substantial radiative recombination of the energy‐transfer‐induced acceptor excitons. Consequently, upon a notable enhancement in the donor‐phase continuity enabled by *n*‐type doping, the charge transport in donor‐dilute devices is significantly facilitated, while the exciton dissociation emerges as the principal efficiency‐limiting step. Meanwhile, a small quantity of N‐DMBI dopants is also found to facilitate exciton dissociation as they can construct desirable type‐II heterojunctions with the doped acceptors and retard interfacial recombination by enlarging D/A interspace.

## 2 Results and Discussion

### 2.1 Acquiring Semi‐Transparency via Donor Dilution

**Figure 1:**
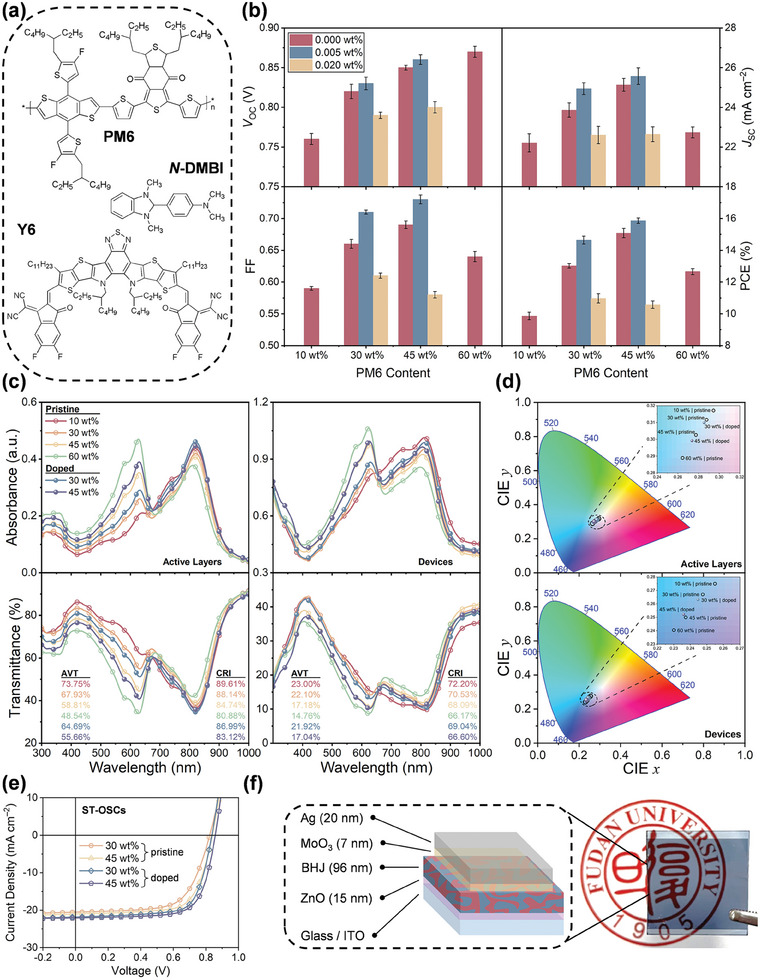
ST‐OSCs based on *n*‐doped donor‐dilute active layers. a) Molecular structures. b) Photovoltaic parameters of PM6(*x* wt.%):Y6:*N*‐DMBI(0, 0.005, 0.02 wt.%) based opaque devices. c) Absorption/transmittance spectra, and the derived d) color coordinates on CIE chromaticity diagrams of pristine and doped active layers and devices. e) *J–V* curves of the champion pristine and doped ST‐OSCs with 30 and 45 wt.% donors. f) Photograph of the ST‐OSCs containing 30 wt.% PM6 donor and 0.005 wt.% *N*‐DMBI dopant.

**Figure** **1**a shows the molecular structures of PM6 donor, Y6 acceptor, and *N*‐DMBI dopant. For donor‐dilute ST‐OSCs, there is always a tradeoff between PCE and AVT. Herein, the opaque solar cells were firstly constructed with an inverted architecture of ITO/ZnO/PM6(*x* wt.%):Y6:N‐DMBI(*y* wt.%)/MoO_3_/Ag(100 nm) to unveil the overall effects of donor dilution and *n*‐type doping on the photovoltaic performance. Figure 1b summarizes the statistics of open‐circuit voltage (*V*_OC_), short‐circuit current (*J*_SC_), fill factor (FF) and PCE that are extracted from their current‐voltage (*J*−*V*) curves (Figure S1, Supporting Information) measured under the stimulated AM1.5G illumination. More details about the champion devices will be discussed in Section 2.4. Note that the mass ratio of PM6:Y6 in a benchmark BHJ device is typically 1:1.2, corresponding to ca. 45 wt.% PM6 in this scenario. Among those pristine solar cells, this optimal composition yields the highest average *J*_SC_ (25.57 ± 0.417 mA cm^−2^) and FF (0.69 ± 0.006) that contribute to the best‐performing PCE (15.08 ± 0.293%) in accordance with the literature values.^[^ ^34^ ^]^ As the donor content is reduced to 30 and 10 wt.%, all four photovoltaic parameters progressively deteriorate, which could be imputed to the less interconnective charge transport networks. It is interesting to note that a higher donor content of 60 wt.% slightly raises the *V*_OC_ from 0.86 ± 0.003 V to 0.87 ± 0.007 V but remarkably decreases *J*_SC_ and FF. Similar results can be found in other research works, while the underlying rationales remain unclear.^[^ ^30^, ^35^ ^]^ For instance, Street and coworkers suggested that the interfacial CTS possessed a D:A averaged electronic structure due to the intermolecular delocalization of electron and hole carriers in the bound state.^[^ ^36^ ^]^ Therefore, it can be speculated that increasing the donor content could generate more high‐energy singlet excitons and elevate the CTS energy^[^ ^35^ ^]^ through hybridization, leading to an increase of *V*_OC_. The photovoltaic performance of the devices comprising 30 and 45 wt.% PM6 donor can be thus further improved by incorporating 0.005 wt.% *N*‐DMBI dopant, which is the optimal doping concentration borrowed from our previous work.^[^ ^26^ ^]^ In specific, such a small amount of dopant was demonstrated to enhance light‐harvesting and charge transport by facilitating the crystallization of both donor and acceptor domains, as well as retard the geminate recombination of CTS by dilating the D/A interspace.^[^ ^26^ ^]^ However, excessive dopant inclusions (i.e., 0.02 wt.%) can destruct the long‐range packing orderness of acceptor molecules, and this is ruinous for charge transport that drastically downgrades *V*_OC_, *J*_SC_, FF, and PCE.^[^ ^26^ ^]^

We then evaluated the optical transparency of donor‐dilute devices. Figure 1c presents the absorption and transmittance spectra for the active layers with various donor dilution and their corresponding semitransparent solar cells obtained by thinning the top Ag electrode down to 20 nm. The absorption of neat PM6 mostly lies within the visible‐light band from 450 to 700 nm featuring absorbance maxima at 623 nm, while the neat Y6 exhibits strong near‐infrared absorption from 600 to 950 nm with a signature peak at 836 nm (Figure S2, Supporting Information). For blend films, with the donor dilution from 60 to 10 wt.%, the peaks of PM6 and Y6 become more red‐ and blue‐shifted, respectively, aside from the linear variation in absorbance. This could be attributed to the varied chemical environment since the acceptor molecules would coordinatively interact with the decreasing number of donor molecules.^[^ ^35^ ^]^

The incorporation of optimal 0.005 wt.% *N*‐DMBI is found to intensify the film absorbance across the whole band, which is attributed to the enhanced crystallinity of both donor and acceptor.^[^ ^26^ ^]^ The companion transmittance spectra of these samples bear the mirrored features of the absorption spectra, from which the metrics of transparency can be derived (Figure 1c,d). Collectively, the donor dilution can incrementally upgrade both AVT and color rendering index (CRI) for the active layers and devices, along with the chromaticity coordinates approaching the white point. These results suggest that the lower donor content allows more visible light to transmit through the active layer with less color distortion, thereby rendering the devices more transparency and color fidelity. For the active layer with medium 45 wt.% PM6, the AVT is barely as low as 58.81% while it can reach 67.93% and 73.75% once the donor content drops to 30 and 10 wt.%, respectively. On their assembled devices, all AVTs become substantially declined, among which the devices with 45 and 30 wt.% PM6 display the lowest values of 17.18% and 22.1%, respectively. However, further reducing the PM6 content to 10 wt.% only results in a marginal AVT ascent of 0.9%. This implies 30 wt.% PM6 is the optimal dilution donor on account of both PCE and AVT. Notably, unlike single active layers, the device AVT is less linearly varied with the donor concentration, and the interlayer optical couplings might play a role in this case.^[^ ^37^ ^]^ In addition, the trace doping with 0.005 wt.% N‐DMBI only leads to the insignificant AVT losses of 0.14% and 0.18% for 45 and 30 wt.% PM6 devices, respectively.

Afterward, both pristine and doped ST‐OSCs were fabricated based on the donor proportion of 30 wt.% and compared with the control device containing conventional 45 wt.% PM6 (Figure 1e). The schematic device architecture with a photograph to visually illustrate the device transparency is provided in Figure 1f. While similar effects of donor dilution and molecular doping on photovoltaic performance are observed for both opaque and semi‐transparent solar cells as discussed above, the thinning of top Ag electrode drastically depresses J_SC_ of the latter (Figure 1b and **Table** **1**). Taking the doped cells with 45 wt.% PM6 as an example, the opaque and semi‐transparent devices are subjected to similar *V*_OC_ (0.86 V) and FF (0.73−0.74), yet their *J*_SC_ of 25.07 and 22.09 mA cm^−2^ lead to a drastic reduction of PCE from 16.3% to 13.89%, respectively. According to the systematic study by Li et al, in addition to a partial optical loss, such a large J_SC_ reduction is predominantly ascribed to the exacerbating surface‐trap‐assisted charge recombination due to the deficient charge extraction by a thinned Ag electrode.^[^ ^38^ ^]^ Remarkably, after doping, the *V*_OC_, J_SC_, and FF of the semi‐transparent device with 30 wt.% PM6 are all improved from 0.82 to 0.84 V, 20.55 to 21.81 mA cm^−2^ and 0.66 to 0.71, thus delivering a prominent increase in PCE from 11.09% to 13.03%, and the highest light utilization efficiency (LUE) of 2.86%.

**Table 1:** Photovoltaic parameters of the pristine and *N*‐DMBI doped (0.005 wt.%) ST‐OSCs based on the PM6(30, 45 wt.%):Y6 blend active layer.

	PM6 wt.%	*V*_OC_ [V]	*J*_SC_ [mA cm^−2^]	FF	PCE [%]	LUE [%]
Pristine	30	0.82	20.55	0.66	11.09	2.45
	45	0.84	20.99	0.70	12.29	2.11
Doped	30	0.84	21.81	0.71	13.03	2.86
	45	0.86	22.09	0.73	13.89	2.37

### 2.2 Visualizing Charge Transport Networks

**Figure 2:**
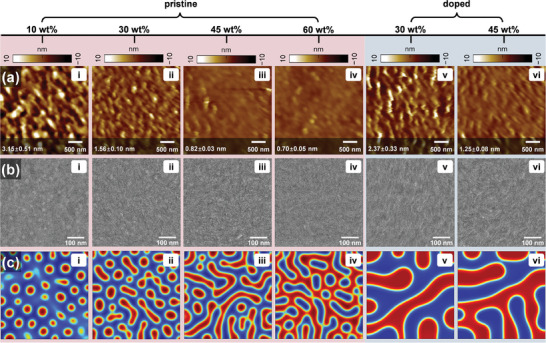
Phase separation and continuity. a) TP‐AFM height images, b) TEM images, and c) 2D phase‐field simulation for the pristine and doped PM6 (red):Y6 (blue) blend films with various PM6 contents.

For the same active layer materials, the construction of charge transport channels and D/A interfaces plays a decisive role in photovoltaic performance. Thus, a series of morphological characterizations were carried out to visualize the effects of *n*‐doping on regulating phase‐separation in donor‐dilute active layers. To start with surface topography, the root‐mean‐square (RMS) roughness of pristine and doped PM6(*x* wt.%):Y6 films was measured by the tapping‐mode atomic force microscopy (TP‐AFM). As shown in **Figure** **2**a‐i−iv, the pristine film containing 10 wt.% PM6 features the most rugged surface and consequently the largest RMS roughness of 3.15 ± 0.51 nm, which is primarily attributed to the self‐aggregation of those abundant highly crystalline acceptors.^[^ ^39^ ^]^ Increasing the donor content to 30, 45, and 60 wt.% can progressively flatten the film surface, which reduces the roughness to 1.56 ± 0.1, 0.82 ± 0.03, and 0.7 ± 0.05 nm, respectively. These results indicate a favorable miscibility between PM6 and Y6, such that the enrichment of PM6 can effectively inhibit the aggregation of Y6. Owing to the enhanced film crystallization, the surface roughness of the doped films containing 30 and 45 wt.% PM6 increases to 2.37 ± 0.33 and 1.25 ± 0.08 nm, respectively (Figure 2a‐v,vi). Transmission electron microscopy (TEM) images follow a similar regularity, but the higher resolution unveils the globular donor agglomerates dispersed in the matrix of acceptor in Figure 2bi, as well as an increasingly homogenized film structures in Figure 2b‐ii−iv. Moreover, unlike the blurred domains shown in pristine films, the doped analogs are textured with curled fibrils (Figure 2b‐v,vi), offering direct evidence for the regulation of phase separation by molecular doping. Foremost, the doped films containing 30 and 45 wt.% PM6 exhibit comparable morphological features, implying an attainable near‐ideal phase separation for donor‐dilute active layers with assistance of *n*‐doping.

In order to quantitatively compare the D:A miscibility before and after *n*‐doping, the contact angle measurement was performed on the films of PM6:*N*‐DMBI (0, 0.017, 0.011 wt.%) and Y6:*N*‐DMBI (0, 0.007, 0.009 wt.%) (Figure S3 and Table S1, Supporting Information). Notably, the predicated doping concentration of 0.005 wt.% was derived in terms of the total blend mass, which is hereby converted with regard to the single component in PM6 (30, 45 wt.%):Y6 films (see Experimental Section). According to the Owens‐Wendt model calculations, the surface energy (*γ*_SV_) of PM6:*N*‐DMBI(*x* wt.%) films remains almost constant (20.37−20.59 mN m^−1^) but increases from 25.82 to 26.41 and 27.69 mN m^−1^ for the Y6 films containing more *N*‐DMBI with a predominant contribution from polar component. The effect of *n*‐doping on strengthening the polarity of Y6 molecules is also reflected in the lowered solubility, whereas there is little change for PM6 (Figure S4, Supporting Information).^[^ ^27^ ^]^ Such differences well exemplify the pinpointed functioning on acceptors by *n*‐doping. By substituting γ_SV,PM6_ and γ_SV,Y6_, the Flory‐Huggins interaction parameter (*χ*) between PM6 and Y6 is evaluated to be 0.29 K in the absence of *N*‐DMBI (see Experimental Section). In the blends with 30 and 45 wt.% PM6, when the concentration of *N*‐DMBI relative to the mass of Y6 rises from 0.007 to 0.009 wt.%; in other words, falls from 0.017 to 0.011 wt.% relative to the mass of PM6, the *χ*_PM6:Y6_ is raised from 0.36 to 0.56 K, suggesting a descended PM6:Y6 miscibility by *n*‐doping. As a result of *n*‐doping, both the lower solubility and the higher *χ* value potentiate the demixing of donor and acceptor during the desolvation of active layer solution, possibly enabling the formation of large and continuous phases even if donor is deficient.

To further substantiate this argumentation, 2D phase‐field simulation was performed to compute the theoretical phase morphologies of the pristine and doped active layers with varied D:A compositions. Given an initially well‐mixed immiscible system, the donor and acceptor components can be described by the function of ϕ, where ϕ  =   ± 1 represents pure phases. The initial state of a mixture is generated when ϕ_*mean*_ =  0 ± 0.05, and the phase separation will be triggered by random perturbation. The algorithm for this kinetic evolution is based on the Canh–Hilliard Equation (1), where ν is mass mobility, λ is free energy density of mixing, ε is interfacial parameter, and Ψ is a function describing the mass transport driven by chemical potential gradient.^[^ ^40^, ^41^ ^]^

(1) $$\frac{\partial \phi }{\partial t}=\nabla \times \frac{\nu \lambda }{\varepsilon^{2}}\nabla \Psi$$

Accordingly, the volume fractions of the two components can be depicted as *V*_1_ = (1 + ϕ) /2 and *V*_2_ = (1 − ϕ) /2, and the term of λ/ε can be roughly correlated with the interfacial tension between donor and acceptor (γ_D: A_) by following Equation (2)^[^ ^42^, ^43^ ^]^:

(2) $$\gamma_{D:A}=\frac{2\sqrt{2}}{3}\;\frac{\lambda }{\varepsilon }$$

The function of Ψ is controlled by Equation (3):

(3) $$\psi \;=\;-\nabla \times \varepsilon^{2}\nabla \phi +\left(\phi^{2}-1\right)\phi$$

During phase separation, the total mass in the system, which is the integral of the phase field variable ϕ about the volume fraction *V_i_*, should be conserved, and the boundary condition of ′wettable interface′ is applied to solve Equation (1)^[^ ^42^ ^]^:

(4) $$\left\{\begin{array}{c} n\cdot \varepsilon^{2}\nabla \phi \;=\varepsilon^{2}\;\cos\left(\theta_{w}\right)\left|\nabla \phi \right|\\ n\cdot \frac{\nu \lambda }{\varepsilon^{2}}\nabla \Psi \;=\;0 \end{array}\right.$$

In this work, a square space of 200 × 200 nm was modeled using COMSOL Multiphysics software (see details in Experimental Section), in which the input variables include the mixture compositions and the contact angle (θ_*w*_) of each component. As shown in Figure 2c‐i−vi, the simulated phase morphologies well resemble the measured TEM images, accordingly. In particular, at 30 wt.% PM6, the isolated small donor domains (red) in the pristine mixture (Figure 2c‐ii) become visibly enlarged and interconnected in the doped counterpart (Figure 2c‐v), which is comparable to the phase configuration in the doped mixture with 45 wt.% PM6 (Figure 2c‐vi). These results once again validate the efficacy of leveraging *n*‐doping to reinforce the bi‐continuity of the charge transport networks in donor‐dilute active layers from the standpoint of theoretical simulation.

**Figure 3:**
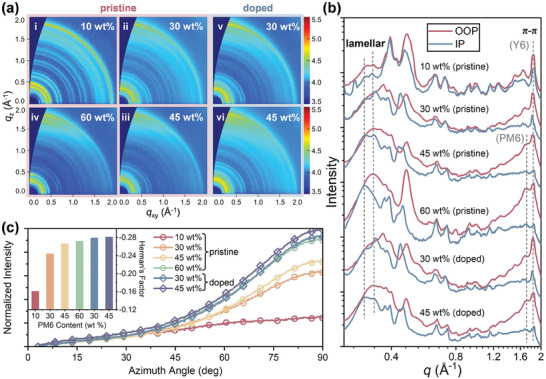
Molecular packings and charge transport patency. a) 2D GIWAXS patterns, b) the corresponding 1D linecuts in both OOP and IP directions, and c) the integrated diffraction intensity of (010) peak as a function of azimuthal angle with respect to *q*_xy_ direction, for the pristine and doped PM6:Y6 blend films with various PM6 contents.

Grazing‐incidence wide‐angle X‐ray scattering (GIWAXS) technique was used to unveil the impact of donor‐dilution and *n*‐doping on the more microscale molecular stacking behavior. **Figure** **3**a‐i−vi provide the 2D diffraction patterns of PM6(10, 30, 45, 60 wt.%):Y6 pristine and PM6(30, 45 wt.%):Y6 doped films, respectively. For all blends, the pronounced diffractions of the π−π stacking (010) in the out‐of‐plane (OOP) direction, as well as the lamellar stacking (100) in the in‐plane (IP) direction suggest a preferential face‐on orientation that favors the vertical charge transport in devices. The diffractograms of single components are also featured with face‐on orientation, where PM6 exhibits stronger lamellar stacking depicted by dispersive spots, while the more prominent *π−π* stacking and multiple relatively uniform diffraction rings are found for Y6 (Figure S5, Supporting Information). These results reveal that the lamellar and π−π stacking diffractions of the blend films predominantly arise from the corresponding PM6 and Y6 domains, and the diffraction features of a certain component could become more distinctive at a higher content. Integrating the 2D patterns in *q*_z_ and *q*_xy_ directions yields the 1D linecuts in Figure 3b, from which the molecular stacking distance (*d*) and crystal coherence length (*L*_C_) can be derived based on the characteristic peak position (*q*) and full‐width‐at‐half‐maxima (Δ*q*). For pristine blend films, when the donor content increases from 10 to 30/45/60 wt.%, there is little change in *d*_π − π_ (≈3.5 Å) while *d*_lamellar_ is enlarged from 18.78 to 19.03/19.15/19.27 Å due to the D:A intermixing. With *N*‐DMBI incorporation, the *d*_lamellar_ is further dilated due to the intercalation of dopant counterions, whereas the *d*_π − π_ is slightly contracted as a result of the strengthened electrostatic attractions between adjacent acceptor molecules after acquiring extra electrons via *n*‐doping.^[^ ^26^ ^]^ Remarkably, the π−π stacking of PM6 molecules can scarcely observed for the pristine films with 30 wt.% donor or less, yet it becomes visibly enhanced upon *n*‐doping albeit weaker than that of the 45 wt.% PM6 film. Compared with pristine films, the doped ones containing 30/45 wt.% PM6 exhibit an $L_{C,\pi -\pi }^{Y6}$ enlargement (i.e., domain size) from 48.2/30.9 to 52.1/37.6 Å, alongside an increase in the Herman's orientation factor (*f*) from −0.24/−0.26 to −0.27/−0.28 (Figure 3c). Collectively, it is evident that the donor dilution suffers from fewer pure phases and weakened hole transport, and meanwhile the excessive acceptors deteriorate the orientation of molecular packings. Nevertheless, *n*‐doping can mitigate this issue in two aspects—by reducing D:A miscibility to promote the self‐aggregation of the diminished donor molecules, and by fostering the epitaxial crystallization of blend films with assistance of the nucleation seeds provided by the dopant molecules of high surface tension and planarity.^[^ ^44^, ^45^ ^]^

### 2.3 Probing Excited State Dynamics

**Figure 4:**
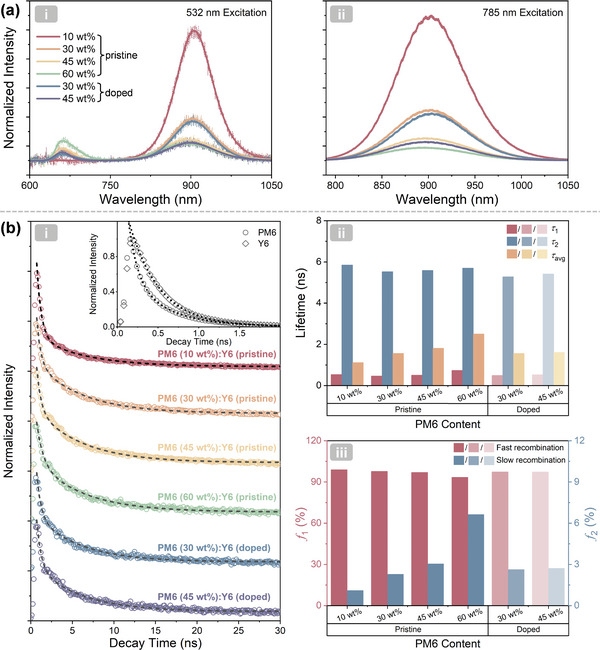
Exciton quenching and lifetime. a) Steady‐state PL spectra of pristine and doped PM6(*x* wt.%):Y6 blend films at i) 785 and ii) 532 nm excitations. b) i) TRPL spectra of pristine and doped PM6(*x* wt.%):Y6 blend films at 438 nm excitation, corresponding ii) exciton lifetimes, and iii) non‐radiative and radiative recombination fractions derived from biexponential decay model.

With the aid of photoluminescence (PL) and transient absorption (TA) spectroscopies, we further rationalize the correlation of phase morphology on the photo‐charge generation and recombination processes. **Figure** **4**a shows the steady‐state PL spectra captured via laser scanning confocal microscopy for both pristine PM6(10, 30, 45, and 60 wt.%):Y6 and doped PM6(30, 45 wt.%):Y6 films. Two excitation wavelengths of 532 and 785 nm were used to selectively excite PM6 and Y6, yielding the characteristic emission peaks at 670 and 910 nm, respectively (Figure S6, Supporting Information). Firstly, the emissions of both PM6 and Y6 can be observed under the excitation at 532 nm (Figure 4a‐i), which is ascribed to the effect of FRET from the photoexcited donor to the acceptor.^[^ ^46^ ^]^ With only 10 wt.% PM6, the corresponding PM6 emission is scarcely discernible, while the Y6 emission is the strongest. This suggests that excitons generated in the diluted donors can be efficiently transformed into acceptor excitons via FRET, while the dissociation of the latter is less effective with insufficient D/A interfaces. The Y6 emission is substantially quenched with the increasing donor content up to the optimal 45 wt.% and then remains almost unchanged when the PM6 content is further increased to 60 wt.%. Such Y6 emission quenching indicates the enhanced dissociation of excitons is populated at Y6 with the increment of PM6 ratio. In addition, we found that *n*‐type doping can slightly facilitate the exciton dissociation, which is ascribed to 1) the smaller exciton binding energy resulting from the more polarized acceptor aggregates,^[^ ^47^ ^]^ 2) the type‐II heterojunctions between *N*‐DMBI^+^ and Y6^•−^,^[^ ^30^ ^]^ and 3) the more efficient exciton diffusion within the enlarged crystalline domains^[^ ^9^ ^]^ (see Section 2.2). Second, similar effects of donor concentration and *n*‐type doping on the PL quenching can be observed when only the Y6 component in the blend films is excited at a wavelength of 785 nm as shown in Figure 4a‐ii.

Time‐resolved photoluminescence (TRPL) decays of the same series films are depicted in Figure 4b‐i. All the TRPL kinetics can be well‐fitted by biexponential function with the components of τ_1_ and τ_2_ that are associated with the processes of fast non‐radiative recombination/energy transfer, and slow radiative/trap‐assisted recombination, respectively.^[^ ^48^ ^]^ Contributions of each process can be evaluated by the weighted fractions (*f*_1_ and *f*_2_) of τ_1_ and τ_2_. We can then calculate the average PL lifetime (τ_avg_) from these two components (for details see Experimental Section). All these fitting parameters are summarized in Figure 4b‐ii,iii. In addition, the TRPL kinetics and fitted lifetimes of neat PM6 and Y6 films are provided in Figure 4b‐i inset and Figure S7 (Supporting Information), respectively. For pristine films, a gradual increase in the donor ratio from 10 to 45 wt.% can moderately increase the volume fraction of D:A intermixing, thus accelerating both the fast non‐radiative recombination/FRET (< 0.5 ns) and the slow radiative/trap‐assisted recombination processes (5−6 ns). As a result, τ_1_ and τ_2_ are slightly shortened from 0.53 and 5.85 ns to 0.5 and 5.59 ns. Interestingly, despite smaller τ_1_ and τ_2_, the corresponding τ_avg_ is oppositely prolonged from 1.11 to 1.81 ns principally due to the increase of *f*_2_ from 1.1% to 3.03%. These results suggest that the much slower radiative and/or trap‐assisted recombinations contribute more to the PL decay, while the faster non‐radiative recombination/FRET processes are reduced, resulting in an overall longer PL lifetime. Given that the donor‐generated excitons can be dissociated through either FRET or interfacial electron transfer process, we postulate that the increased number of donor‐acceptor interfaces arising from a more balanced donor ratio enhances the latter process while weakening the former.^[^ ^49^, ^50^ ^]^ For the pristine film with 60 wt.% PM6, the τ_1_, τ_2_ and *f*_2_ are fitted to be 0.73 ns, 5.7 ns and 6.64%, respectively, giving rise to the longest τ_avg_ of 2.5 ns, which implies inefficient exciton dissociation. With special regards to the film containing 30 wt.% PM6, *n*‐doping seems to barely affect τ_1_ and τ_2_ but remarkably raise the *f*_2_ from 2.28% to 2.63%, thereupon endowing it with similar TRPL responses (i.e., τ_avg_) as that of the 45 wt.% PM6 analogs.

**Figure 5:**
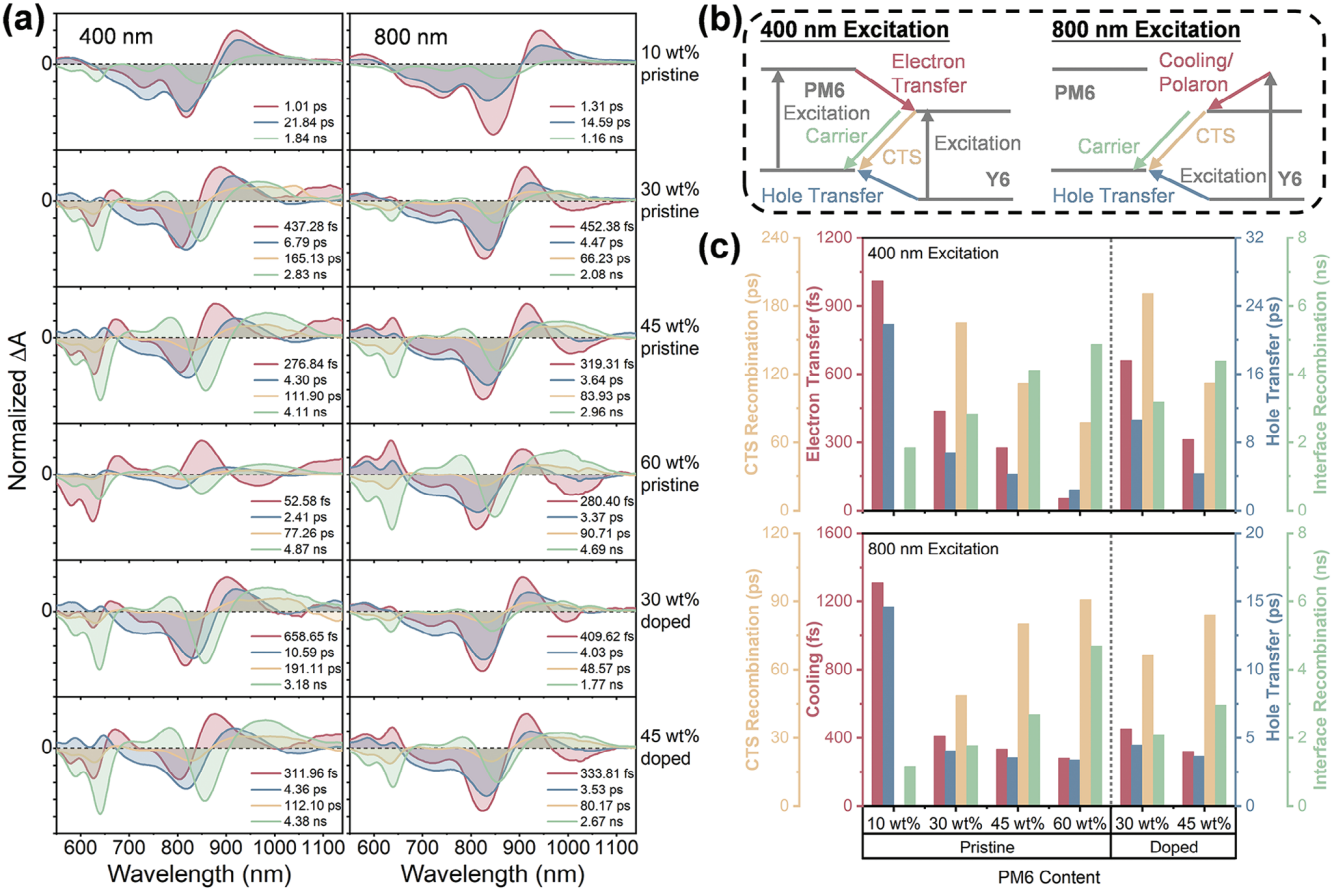
Charge transfer and recombination after photoexcitation. a) Normalized single‐value‐decomposition analysis for the measured TA spectra of pristine and doped PM6(*x* wt.%):Y6 blend films, and the corresponding b) schematics and c) times taken for the processes of hot charge cooling, charge transfer, charge‐transfer state recombination and geminate recombination under the 400 and 800 nm excitations, respectively.

However, the lifetimes of fast component τ_1_ (∼0.5 ns) are limited by the response function of the TRPL setup. We then characterized the ultrafast excited state dynamics in pristine and doped PM6(*x* wt.%):Y6 blend films by TA measurement. The pump pulses with the wavelength of both 400 and 800 nm were used to excite PM6 and Y6, respectively, which allows to identify the pathways for electron and hole transfer. The measured TA spectra are shown in Figure S8 (Supporting Information), and we then analyze the excited state depopulation pathways by the singular‐value‐decomposition (SVD) fittings as shown in **Figure** **5**a. Detailed assignments for each lifetime component have been well documented in our previous work,^[^ ^26^ ^]^ as well as in literature.^[^ ^51^, ^52^ ^]^ For 400 nm excitation, the fastest component (red color) with a lifetime in hundreds of femtoseconds is associated with the electron transfer from PM6 to Y6, characterized by the ground‐state bleaching (GSB) at ≈623 nm and the excited‐state absorption (ESA) at ≈880 nm, which indicate the concurrent occurrence of charge depopulation in PM6 and population in Y6. Similarly, the GSB at ∼836 nm and the ESA at ∼645 nm for the second fastest component (blue color) of a few picoseconds is indicative of hole transfer from Y6 to PM6. In spite of the fact that 400 nm pulse cannot directly excite Y6, excitons can still appear in it as a consequence of energy transfer from donor to acceptor. Another component of 77.3−191.1 ps (yellow color) and the longest one (green color) show dual GSB signals for the excited states of PM6 and Y6, yet the former is more blue‐shifted and weakened, which can be assigned to the CTS recombination and interfacial GR recombination of separated charge carriers, respectively. The assignment of lifetime components is almost similar in the cases of 800 nm excitation. However, since the energy transfer from Y6 to PM6 is energetically unfavored, the electron transfer shall not occur in this scenario. Instead, the fastest component with the lifetime of hundreds of femtoseconds (red color) should be ascribed to the ultrafast process, either hot carrier cooling or polaron formation, prior to the population of the lowest excited states of Y6.^[^ ^53^ ^]^ Figure 5b provides an illustration of these photophysical processes, and Figure 5c summarizes the specific times taken for each process.

For both 400 and 800 nm excitations, the increasing donor content from 10 to 60 wt.% in pristine films can accelerate the electron and hole transfers at D/A interfaces (red and blue bars in Figure 5c), as well as hot carrier cooling (red bars in Figure 5c). Combined with the AFM and TEM images in Figure 2a,b, it is evident that the D:A intermixing can be significantly enhanced with the incorporation of more PM6, primarily attributing to the more flexible molecular structure and the amorphous nature of polymeric donors. As a result, the increases in D/A contacts and energetic disordering expedite the interfacial charge transfer and the hot carrier relaxation,^[^ ^54^ ^]^ respectively. In addition, with the increasing donor content, the CTS lifetimes are found to be shortened/prolonged under 400/800 nm excitations. According to the research by Street and coworkers, the optoelectronic properties of CTS actually reflected the interfacial structure and compositions of donor and acceptor due to the delocalization of the constituent electrons and holes.^[^ ^36^, ^55^ ^]^ Therefore, we propose that the variations in the CTS lifetimes may be associated with the density of singlet excitons versus charge‐transfer excitons at different excitation conditions. In particular, at 400 nm excitation, with the increasing PM6 content, there is a higher density of singlet excitons residing at PM6 without being transferred to Y6, as evidenced by the presence of PM6 emission in the steady‐state PL spectra (Figure 4a). Such singlet excitons can interact with the charge transfer excitons at the D/A interface to form the local excited states (LEs). It was reported that LEs and CTS with similar energy levels are likely to hybridize via electronic coupling.^[^ ^56^, ^57^ ^]^ Therefore, the more short‐lived LEs, the more LE‐CTS hybridization/intermixing, which will inevitably shorten the CTS lifetime. At 800 nm excitation, no LEs are supposed to be formed. The CTS lifetimes are elongated^[^ ^52^ ^]^ with the increasing PM6 content. The same trend can be found for the geminate recombination for both 400 and 800 nm excitations. Such decelerated recombination of separated charge carriers at D/A interface with an increase of PM6 content can be ascribed to the lower dielectric constant (ε_r,PM6_ < 2)^[^ ^58^ ^]^ and larger intermolecular distances (see Section 2.2 for details) of PM6 in comparison to those of Y6 (ε_r,Y6_≅3.5),^[^ ^59^ ^]^ both of which hinder the electronic coupling between electrons and holes at interfaces. For the doped films of PM6(30, 45 wt.%):Y6, the same but slower processes can be assigned from their SVD fittings. For each process, such retardation can be explained as follows: 1) the intercalation of *N*‐DMBI^+^ counterions can expand the spatial distance of D/A interface as evidenced by the enlargement of *d*_lamellar_, resulting in the slower electron/hole transfer as well as the suppressed CTS/carrier recombination at interfaces; and 2) extra electrons donated by *n*‐doping can flatten the energetic landscape via trap‐filling,^[^ ^60^ ^]^ thus decelerating the hot carrier cooling. Besides, the doping‐enhanced film crystallinity is also conducive for exciton diffusion and charge transportation,^[^ ^9^ ^]^ which could play additional roles in prolonging the excited‐state lifetime as revealed by both TRPL and TA measurements.

### 2.4 Correlating with Device Performance

**Figure 6:**
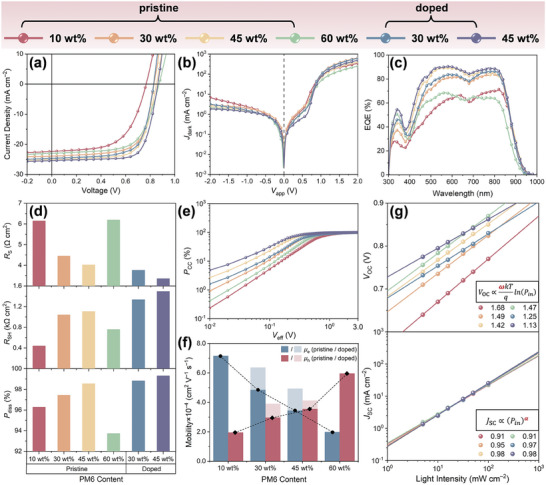
Photo‐electrical characteristics. a) Light *J–V* curves, b) dark *J–V* curves, c) EQE spectra, d) series resistance (*R*_S_), shunt resistance (*R*_SH_), and exciton dissociation probability (*P*_diss_), e) charge collection efficiency (*P*_CC_), f) SCLC‐measured electron (*µ*_e_) and hole (*µ*_h_) mobilities, and g) light‐intensity‐dependent *V*_OC_ and *J*_SC_ of the champion pristine and doped devices based on PM6(*x* wt.%):Y6 blends.

From the discussions above, despite a limited improvement in exciton dissociation, *n*‐type doping is known to reinforce the donor phase continuity and mitigate the interfacial CTS/carrier recombination in the donor‐dilute active layers. Returning to devices, the overall charge transport properties will be investigated in this section as the final building block to further rationalize the correlation between film morphology, photophysics, and photovoltaic performance. Effects of donor proportion and *n*‐type doping on the photovoltaic performance have been specifically discussed in Section 2.1, in which the statistics of *V*_OC_, *J*_SC_, FF, and PCE of the opaque devices as mentioned above are presented in Figure 1b. **Table** **2** summarizes the photovoltaic parameters for the champion opaque devices that are the most outstanding for the same batches of the samples shown in Figure 1b and Figure S1 (Supporting Information). Notably, despite a relatively small PCE enhancement ratio of ≈5.2% (Table 2) for the opaque donor‐dilute champion device after being *n*‐doped, the corresponding average PCE is improved by ≈12.4% (Figure S1, Supporting Information), which is more prominent in comparison to the opaque devices with standard D:A compositions and also consistent with the PCE variations for the semi‐transparent analogs as discussed in Section 2.1. These results indicate the more effective *n*‐type doping in donor‐dilute OSCs, which is attributable to an increasing number of the interactive acceptors. With regards to the champion cells, their light and dark *J*–*V* curves, as well as the external quantum efficiency (EQE) profiles are given in **Figure** **6**a−c, respectively. From light *J*–*V* curves, the series (*R*_S_) and shunt resistance (*R*_SH_) can be estimated from the reciprocal of the slopes at *V*_OC_ and *J*_SC_, respectively. The dependence of photocurrent (*J*_ph_ =  *J* − *J*_dark_) on effective voltage (*V*_eff_ = *V*_app_  − *V*_comp_) is plotted in Figure S9 (Supporting Information), where *V*_app_ is applied voltage, and *V*_comp_ is the compensation voltage at *J*_ph_ =  0. The exciton dissociation probability (*P*_diss_) and charge collection probability (*P*_CC_) can be quantified by the ratios of *J*_SC_/*J*_sat_ and *J*_ph_/*J*_sat_, respectively, where *J*_sat_ refers to the reversed saturation current at *V*_eff_ > 2 V.^[^ ^61^ ^]^ Calculation results are summarized in Figure 6d,e.

**Table 2:** Photovoltaic parameters of the champion solar cells based on the PM6:Y6 blend with various donor content.

	PM6 [wt.%]	*V*_OC_ [V]	*J*_SC_ [mA cm^−2^]	*J*_cal_ [mA cm^−2^]	FF	PCE [%]
Pristine	10	0.77	22.37	21.60	0.59	10.12
	30	0.83	23.94	23.14	0.68	13.54
	45	0.85	25.07	24.71	0.71	15.13
	60	0.87	22.99	22.23	0.65	12.94
Doped	30	0.83	24.64	24.36	0.70	14.24
	45	0.86	25.62	25.28	0.74	16.30

For pristine OSCs based on PM6(*x* wt.%):Y6 active layers, the *R*_S_ firstly drops from 6.14 to 4.44 and 4.01 Ω cm^2^ with an increase of donor content from 10 to 30 and 45 wt.%, followed by a rebound to 6.19 Ω cm^2^ when the PM6 fraction is further raised to 60 wt.%. The *R*_SH_ is varied in an opposite trend, where the PM6 content of 10, 30, 45, and 60 wt.% yields the *R*_SH_ of 0.44, 1.04, 1.11, and 0.76 kΩ cm^2^, respectively. According to the equivalent circuit model, FF of a solar cell is negatively correlated with *R*_S_ while positively correlated with *R*_SH_,^[^ ^62^ ^]^ and these results are well aligned with the corresponding FF listed in Table 2. *R*_S_ is generally a sum of the bulk resistance of functional layers, the interlayer contact resistance, and the probe resistance, while *R*_SH_ is associated with the current leakage that is typically originated from the edge effect and structural defects.^[^ ^63^ ^]^ In Section 2.1, the device containing 10 wt.% PM6 is revealed to possess the roughest surface, which can be correlated with the highest dark current (Figure 6b) and the consequentially lowest *R*_SH_. Besides the enhancement in face‐on orientation, increasing donor content up to 45 wt.% also consolidates the π‐stacks of PM6 molecules, yet the excessive donors (i.e., 60 wt.%) weakens the molecular stacking of Y6 acceptors due to their high miscibility. Moreover, the crystallinity/continuity of both donor and acceptor phases can be strengthened with the assistance of a small amount of *N*‐DMBI dopants. Serving as the channels for charge transportation, these phase characteristics significantly affect carrier mobility and thus *R*_S_, as evidenced by the SCLC measurement (Figure 6f). As for the devices with 45 wt.% PM6, the hole mobility (µ_h_) of 3.55 × 10^−4^ cm^2^ V^−1^ s^−1^ is found to be slightly higher than the electron mobility (µ_e_) of 3.43 × 10^−4^ cm^2^ V^−1^ s^−1^, but their ratio (µ_h_/µ_e_) of 1.03 is the closest to the unity, hinting at the most balanced charge transport. Benefiting from the more continuous charge transport networks and the *n*‐type doping effect on acceptors, the corresponding µ_h_ and µ_e_ are uplifted to 4.93 × 10^−4^ and 4.12 × 10^−4^ cm^2^ V^−1^ s^−1^ with the introduction of *N*‐DMBI dopant, respectively. Despite a concurrent enhancement in µ_h_ from 2.96 × 10^−4^ to 3.9 × 10^−4^ cm^2^ V^−1^ s^−1^ and µ_e_ from 4.84 × 10^−4^ to 6.37 × 10^−4^ cm^2^ V^−1^ s^−1^ for the 30 wt.% PM6 devices upon *n*‐doping, the ratio of µ_h_/µ_e_ is found to drop from 0.76 to 0.61. Overall, *R*_S_ is highly dependent on the phase morphology that determines the charge carrier mobility, for which the undesirably large *R*_S_ can be ascribed to the unbalanced µ_h_/µ_e_ in donor‐dilute devices. Although *n*‐type doping can hardly resolve this issue, it still can reduce *R*_S_ by promoting the absolute values of µ_h_ and µ_e_.

As shown in Figure S9 (Supporting Information), the *J*_ph_ can reach a saturation at a lower *V*_eff_ for the devices with the donor content closer to 45 wt.%, indicative of the less field‐dependent charge transport and the higher *P*_CC_ (Figure 6e). Moreover, the *P*_diss_ of the pristine cells containing 10/30/45/60 wt.% PM6 and the doped cells containing 30/45 wt.% PM6 are also derived to be 96.28/97.44/98.54/93.73% and 98.81/99.30%, respectively, from the *J*_ph_–*V*_eff_ curves, which are consistent with the qualitative analysis of PL and TRPL results in Section 2.3. In addition to the contact structure and the energetic barrier at interfaces, the absolute µ_h_ and µ_e_ as well as their balance are also crucial for charge collection. In other words, if the charge carrier mobility is neither low nor unbalanced, the resulted space charge accumulation can counteract the drift current, thereby lowering *J*_SC_ and FF.^[^ ^63^ ^]^ Given the light‐intensity (*P*_in_) dependences of *V*_OC_ and *J*_SC_ in Figure 6g, diluting donor content is found to deviate the constants of *ω* and *α* in the relations of *V*_OC_∝*ω*kT/q*ln*(*P*_in_) and *J*_SC_∝(*P*_in_)^α^ from unity. The larger *ω* implies the more trap‐assisted and/or surface recombinations, while the *α* is associated with the more bimolecular recombination. With the assistance of *n*‐type doping, the device containing only 30 wt.% PM6 shows a decrease in ω from 1.49 to 1.25 and an increase in α from 0.95 to 0.97, indicating fewer electronic/morphological defects and more efficient charge carrier transport. As shown in Table 2 and Figure S10 (Supporting Information), the current densities (*J*_cal_) derived from the EQE integrals in Figure 6c are well consistent with the corresponding *J*_SC_, reconfirming the reliability of *J*–*V* measurement. Apparently, the photoresponse of the donor‐dilute (i.e., 30 wt.% PM6) and donor‐rich (i.e., 60 wt.% PM6) devices are less efficient within the donor and acceptor bands, respectively, which determines the current loss in the views of light‐harvesting. Considering the proportional reduction in light absorbance due to the lowered donor/acceptor concentration (Figure 1c), it is inferred that the EQE decrease is primarily ascribed to the less efficient exciton dissociation and charge carrier transport.

## 3 Conclusion

In summary, *N*‐DMBI dopants were incorporated into the active layer of donor‐dilute ST‐OSCs to reinforce the donor‐phase continuity. This is enabled by reducing the D:A miscibility through delicately strengthening the acceptor polarity and the self‐aggregation of D/A components. As a result, despite a decrease in donor content from the standard 45 wt.% to the optimal 30 wt.%, the charge transport and collection in the *n*‐doped donor‐dilute ST‐OSCs are comparably efficient. Therefore, besides the device AVT enhancement from 17.18% to 22.1%, the corresponding PCE has been significantly improved from 11.09 to 13.03% compared to the undoped donor‐dilute device. Our investigation also reveals that the exciton dissociation via FRET mechanism becomes less efficient with the decreasing donor content, wherein the energy‐transfer‐induced acceptor excitons can decay radiatively without sufficient D/A interfaces. *N*‐type doping can mitigate this issue by offering acceptors additional energy offset to assist exciton dissociation, while the enlargement of D/A interspace with dopant intercalation is also demonstrated to retard the CTS/charge recombination. By utilizing a series of morphological characterizations coupled with phase‐field simulation, this study experimentally and theoretically unravels the functioning mechanisms of *n*‐type doping on modulating phase separation in BHJ active layers, thus providing insights into the significance of phase continuity and offering an effective strategy to enhance it for boosting the efficiency of donor‐dilute ST‐OSCs.

## 4 Experimental Section

### Materials

PM6 and Y6 were purchased from Nanjing Zhiyan Technology Co. Ltd. (China). *N*‐DMBI was purchased from Sigma–Aldrich. All the materials were used as received without further purification.

### Solubility Testing

Excessive PM6 and Y6 were dissolved in CF solvent with varying concentrations of *N*‐DMBI dopant. It should be noted that the *N*‐DMBI concentrations used here do not correspond directly to their actual content in the film or device samples. Instead, they are employed solely for qualitative assessment of *n*‐type doping effect on PM6/Y6 solubilities. All the solutions were stirred at room temperature for 2 h, then heated to 85 °C for 5 min to the supersaturated solutions and activate *N*‐DMBI doping. After cooling to room temperature, stirring continued for 2 h, followed by overnight settling to precipitate the excessively dissolved chemicals. The dilute saturated solutions with unknown concentration were prepared by diluting a small amount of the supernatant 3000‐folds. A series of CF solutions of PM6 and Y6 were prepared with known concentrations ranging from 0.002 to 0.01 mg mL^−1^. Optical absorption spectra were acquired for all solutions, and unknown concentrations of dilute saturated solutions were estimated from the linear fittings of absorbance maxima according to the Beer‐Lambert law. Finally, PM6 and Y6 solubilities were estimated as 3000 times the unknown concentrations.

### Device Fabrication and Measurement

The ITO coated substrates (15 Ω sqr^−1^) were ultrasonically washed with detergent, deionized water, acetone, and isopropanol in sequence for 20 min each step. After drying with nitrogen flux, the substrates were treated with UV‐ozone for 15 min. All solar cells were fabricated in the architecture of ITO/ZnO/PM6(*x* wt.%):Y6:*N*‐DMBI(0, 0.005 wt.%)/MoO_3_/Ag. The 25 mg mL^−1^ 2‐methoxyethanol solution of zinc acetate dihydrate with 0.75 vol% 2‐aminoethanol was spin‐coated onto the clean substrates in air at 3000 rpm for the deposition of ZnO electron transport layer, which were then thermally annealed at 200 °C for 1 h. To deposit active layers, the substrates were then transferred into an argon‐filled glovebox, and the solutions of PM6(10, 30, 45, 60 wt.%):Y6:N‐DMBI(0, 0.005 wt.%) were spin‐coated onto the rotating substrate at 3000 rpm, followed with the thermal annealing at 85 °C for 10 min. Finally, the hole transport layer of 7 nm MoO_3_ and the top electrode of 150 nm (for opaque devices) or 20 nm (for semi‐transparent devices) Ag thin films were thermally evaporated onto the samples at a base pressure of 10^−4^ Pa, which were patterned with a shadow mask. Unencapsulated solar cells with an active area of 0.04 cm^2^ were tested under AM 1.5G irradiation (Newport‐Oriel) in air, and the *J*–*V* curves were recorded using a Keithley 2400 source meter, with light intensity determined by a 2 × 2 cm^2^ standardized monocrystalline silicon cell. *J*–*V* curves were scanned with a voltage increment of 0.02 V and a dwell time of 20 ms. EQE profiles were measured using the QE‐R setup designed by Enli Technology Co. Ltd with 500 W xenon lamp.

### SCLC Characterization

Electron‐ and hole‐only devices were fabricated with configurations of ITO/ZnO/PM6(10, 30, 45, 60 wt.%):Y6:*N*‐DMBI(0, 0.005 wt.%)/PNDIT‐F3N/Ag and ITO/PEDOT:PSS/PM6(10, 30, 45, and 60 wt.%):Y6:*N*‐DMBI(0, 0.005 wt.%)/MoO_3_/Ag, following a procedure similar to that of the solar cells, with the exception of PNDIT‐F3N and PEDOT:PSS layer depositions. Methanol solution (0.5 mg mL^−1^) of PNDIT‐F3N with 0.5 vol% acetic acid was spin‐coated onto the active layer at 3000 rpm for 40 s, followed by annealing at 85 °C for 5 min. The water solution of PEDOT:PSS was used as received, which was spin‐coated onto substrates at 3000 rpm for 40 s and annealed at 150 °C for 20 min in air. SCLC *J*–*V* curves were measured in dark with a Keithley 2400 source meter. Charge mobilities (*µ*) were determined from linear fittings of the semi‐log plot of SCLC curves according to the Mott–Gurney model of $J\;=\frac{9}{8}\;\mu \varepsilon_{r}\varepsilon_{0}{\frac{V}{d^{3}}}^{2}$, where *J* is the current density, *V* is the applied voltage, ε_r_ is the relative dielectric constant of 3 for organic semiconductors, ε_0_ is the vacuum permittivity of 8.8542 × 10^−12^ F m^−1^, and *d* is the active layer thickness measured by step‐profilometer.

### Film Morphology

For AFM imaging, the samples of ZnO/PM6(10, 30, 45, and 60 wt.%):Y6:*N*‐DMBI(0, 0.005 wt.%) were prepared on the Si substrate following the same procedures as what applied for the deposition of device active layers, and the measurement was performed using NX10 microscope from Park Systems Co. Ltd. in tapping mode. To prepare TEM samples, the same blend films on Si substrate were immersed into the non‐orthogonal solvent of methanol, and the floated films were then transferred onto the copper mesh. The TEM images were captured by the equipment of JEOL JEM‐F200. For GIWAXS measurement, samples were drop‐casted on the Si substrate from the blend solutions, and the 2D diffraction patterns were acquired by beamline BL14B1 at SSRF with an X‐ray source of 10 keV and incident angle of 0.2°, and the measured diffraction patterns were further corrected by a MATLAB toolbox of *GIXSGUI*.^[^ ^64^ ^]^ To calculate Herman's orientation factor, the operator given in the main text could be determined by the equation of ${\left\langle cos^{2}\phi \right\rangle }_{hkl}=\frac{\int_{0}^{\pi /2}I\left(\phi \right)cos^{2}\phi \sin\phi \;d\phi }{\int_{0}^{\pi /2}I\left(\phi \right)\sin\phi \;d\phi }\;$, wherein ϕ is Azimuthal angle and *I*(ϕ) is the scattered intensity. The phase‐field simulations were carried out by COMSOL Multiphysics package.

### Contact Angle Measurement

For PM6:Y6 blend films, the *N*‐DMBI dopant concentration (*x* wt.%) is calculated as $x_{blend}\;=\frac{m_{N-\mathrm{DMBI}}}{m_{\mathrm{PM}6}+m_{Y6}}\;$ and $x_{\mathrm{PM}6\;\mathrm{or}\;Y6}\;=\frac{m_{N-\mathrm{DMBI}}}{m_{\mathrm{PM}6\;\mathrm{or}\;Y6}}\;$, wherein *m_i_* are denoted the masses of *N*‐DMBI, PM6, and Y6. Given the dopant concentration (*x_blend_*) of 0.005 wt.% in the blends with 30 and 45 wt.% PM6, the corresponding *x*_PM6_ and *x*_Y6_ for neat PM6 and Y6 are calculated to be 0, 0.017, 0.011 wt.% and 0, 0.007, 0.009 wt.%, respectively. These solutions were then spin‐coated onto the Si substrates at 3000 rpm followed with 10 min thermal annealing at 85 °C. The contact angle images were captured by Dataphysics‐OCA25, and the two liquids of deionized water (H_2_O) and ethylene glycol ((CH_2_OH)_2_) with known $\gamma_{LV}^{d}$/$\gamma_{LV}^{p}$ of 22.1/50.7 and 29.3/19.0 mN m^−1^, respectively, were used for the determination of surface tensions according to the Owens‐Wendt model of $\gamma_{LV}\;\left(1+cos\theta \right)=\;2\sqrt{\gamma_{SV}^{d}\gamma_{LV}^{d}}+2\sqrt{\gamma_{SV}^{p}\gamma_{LV}^{p}}$ and $\gamma_{SV}=\gamma_{SV}^{d}\;+\gamma_{SV}^{p}$.^[^ ^65^ ^]^ The Flory‐Huggins interaction parameters were calculated according to the equation of $\chi \;=\;K{\left(\sqrt{\gamma_{SV,X}}+\sqrt{\gamma_{SV,Y}}\right)}^{2}$, wherein *K* is the proportionality constant.^[^ ^40^ ^]^

### Optical Spectra

Steady‐state absorption spectra and PL spectra were measured by UV–Visible spectrometer of Agilent 8453 (Agilent Technologies Inc.) and the laser scanning confocal microscope of inVia03040404 (Renishaw Co. Ltd.), respectively. TRPL spectra were measured by SpexFluorolog 1681 spectrofluorometer with the excitation wavelength of 438 nm. The TRPL spectra were fitted with the biexponential function of $I\;=I_{0}\;+A_{1}e^{-t/\tau_{1}}+A_{2}e^{-t/\tau_{2}}$, the average exciton lifetime is calculated as $\tau_{\mathrm{avg}}=\frac{A_{1}\tau_{1}+A_{2}\tau_{2}}{A_{1}+A_{2}}\;$, and the contributions of the fast and slow processes are evaluated by $f_{i}=\frac{A_{i}/\tau_{i}}{A_{1}/\tau_{1}+A_{2}/\tau_{2}}\;$. TA spectra were recorded by a femtosecond pump‐probe setup in nitrogen atmosphere. The laser sources of 400 and 800 nm with 80 fs pulse length were used to selectively excite PM6 and Y6, and the charge generation process was analyzed by SVD method using Glotaran software package.

## Conflict of Interest

The authors declare no conflict of interest.

## Author Contributions

J.X. conducted the experiments and wrote the original paper under the supervision of Z.L. W.L. and K.Z. performed TRPL and TA measurements. K.Z. supervised W.L. and assisted in writing the manuscript. Z.L. provided a guide to the manuscript and led the project. All authors discussed the results and commented on the manuscript.

## Supporting information

Supporting Information

## Data Availability

The data that support the findings of this study are available from the corresponding author upon reasonable request.
