# Supplementary material for: N‐Doping Donor‐Dilute Semitransparent Organic Solar Cells to Weaken Donor: Acceptor Miscibility and Consolidate Donor‐Phase Continuity
Source: Adv Sci (Weinh). 2024 Jun 17;11(31):2404135. doi: 10.1002/advs.202404135 (PMC11336925; doi:10.1002/advs.202404135)
Supplement: Supplementary file 1 — Supporting Information [file ADVS-11-2404135-s001.docx]

Supporting Information

*N*-Doping Donor-Dilute Semitransparent Organic Solar Cells to Weaken Donor:Acceptor Miscibility and Consolidate Donor-Phase Continuity

Jiaqi Xie,^1^ Weihua Lin,^2^ Kaibo Zheng,^2,3*^ and Ziqi Liang^1,4*^

[*]^1^Prof. Z. Liang, J. Xie

Department of Materials Science

Fudan University

Shanghai 200433, China

Email: zqliang@fudan.edu.cn

^2^Dr. K. Zheng, W. Lin

Department of Chemical Physics and NanoLund

Lund University, Box 124, 22100

Lund, Sweden

[*]^3^Prof. K. Zheng

Department of Chemistry

Technical University of Denmark

DK-2800 Kongens Lyngby, Denmark

Email: kzheng@kemi.dtu.dk

[*]^4^Prof. Z. Liang

Institute of Optoelectronics

Fudan University

Shanghai 200433, China


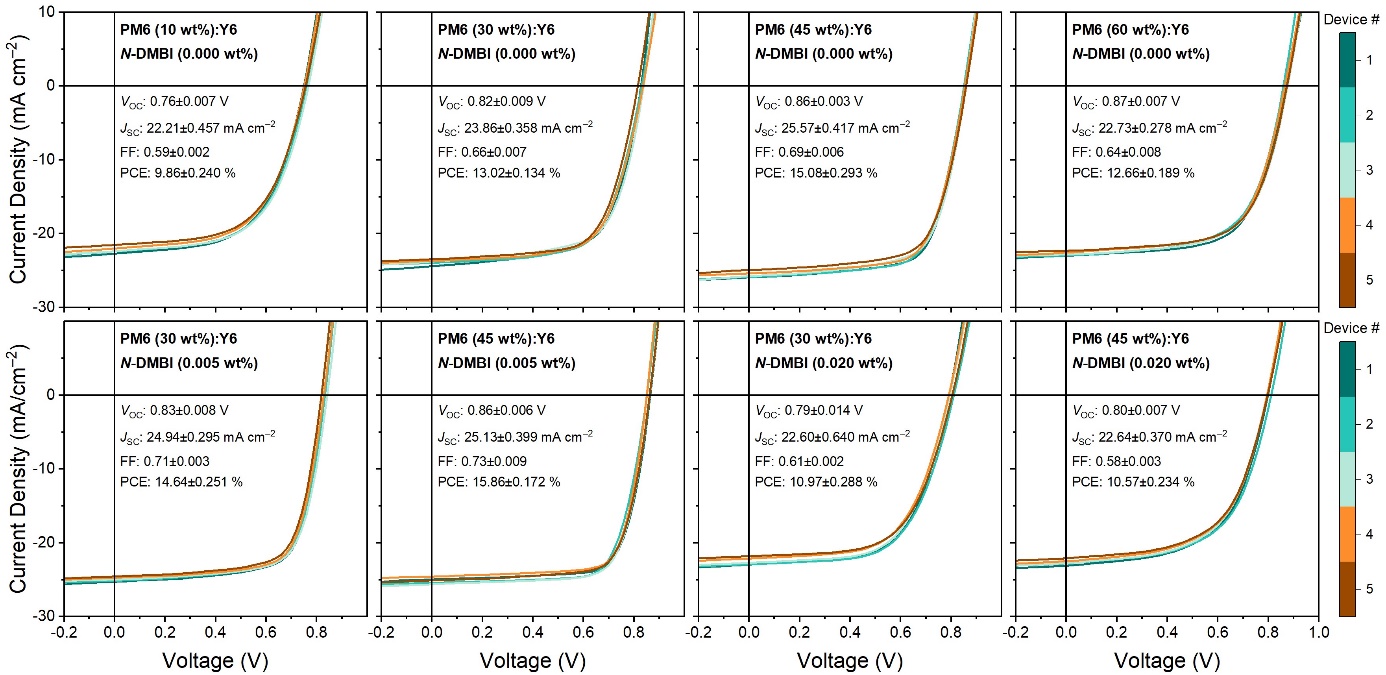


**Figure S1.** Device reproducibility: light *J*–*V* curves of the opaque solar cells based on PM6(*x* wt%):Y6:*N*-DMBI(*y* wt%).


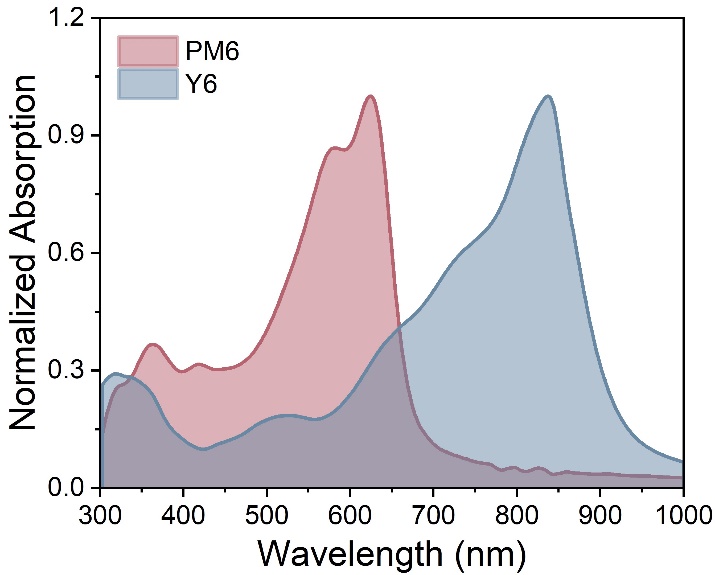


**Figure S2.** Normalized optical absorption spectra of neat PM6 and Y6 thin films.


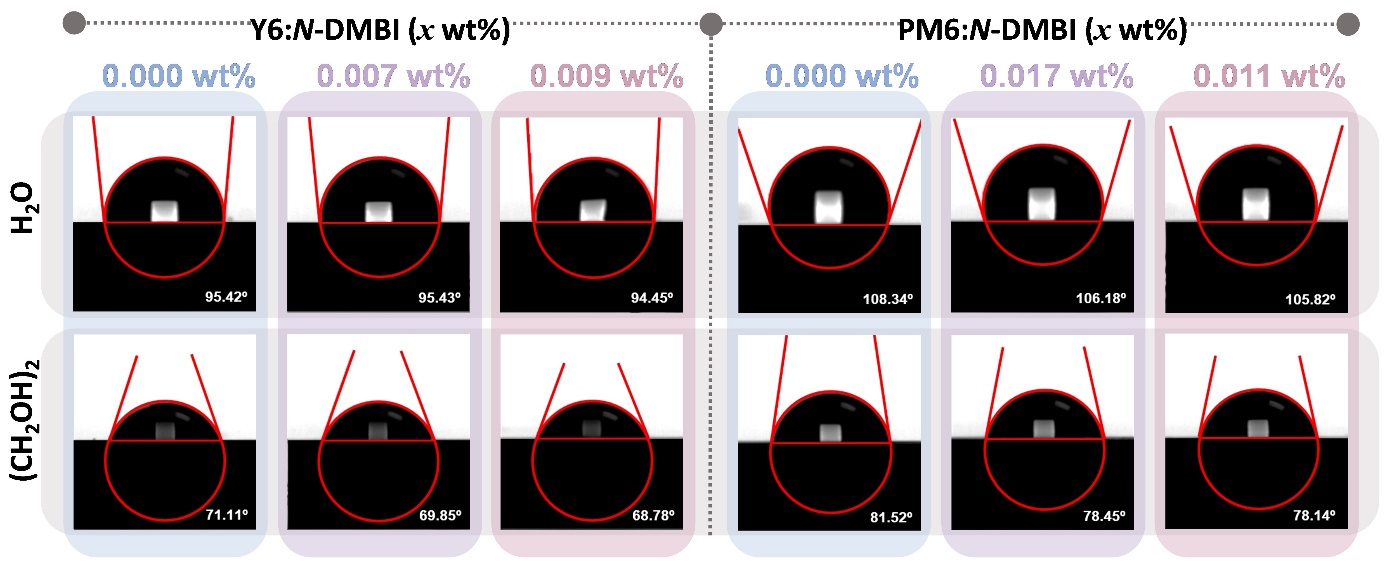


**Figure S3.** Contact angle measurement. Photographs of water (H_2­_O) and ethylene glycol ((CH_2_OH)_2_) droplets on the surface of Y6:*N*-DMBI(0, 0.007, 0.009 wt.%) and PM6:*N*-DMBI(0, 0.017, 0.011 wt.%) films deposited on glass substrates.

**Table S1.** Surface energy of Y6:*N*-DMBI(0, 0.007, 0.009 wt.%) and PM6:*N*-DMBI(0, 0.017, 0.011 wt.%) films determined based on the results of contact angle measurement.

|  | ***N*-DMBI (wt%)** | $\boldsymbol{\gamma}_{\mathbf{SV}}^{\mathbf{d}}$ **(mN m^−1^)** | $\boldsymbol{\gamma}_{\mathbf{SV}}^{\mathbf{p}}$ **(mN m^−1^)** | $\boldsymbol{\gamma}_{\mathbf{SV}}$ **(mN m^−1^)** |
| --- | --- | --- | --- | --- |
| **Y6 Films** | 0.000 | 25.38 | 0.44 | 25.82 |
|  | 0.007 | 25.36 | 1.05 | 26.41 |
|  | 0.009 | 25.42 | 2.27 | 27.69 |
| **PM6 Films** | 0.000 | 19.96 | 0.56 | 20.52 |
|  | 0.017 | 20.28 | 0.31 | 20.59 |
|  | 0.011 | 20.26 | 0.11 | 20.37 |


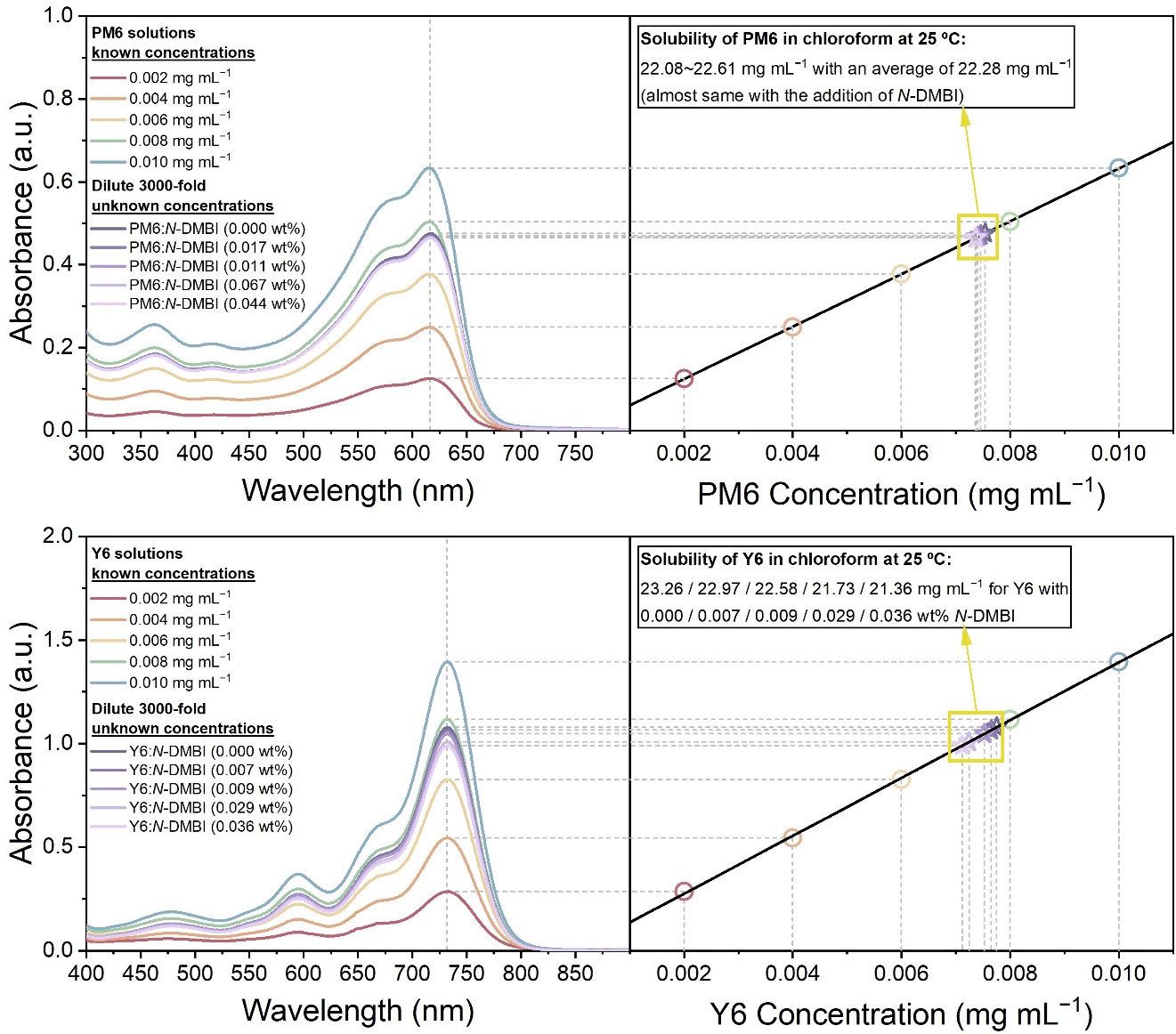


**Figure S4.** Solubility testing. Absorption spectra of the chloroform solutions of PM6 and Y6 with a set of known concentrations from 0.002 to 0.01 mg mL^–1^, as well as the unknown concentration of the PM6/Y6:*N*-DMBI(*x* wt%) solutions that are diluted by 3000-folds.


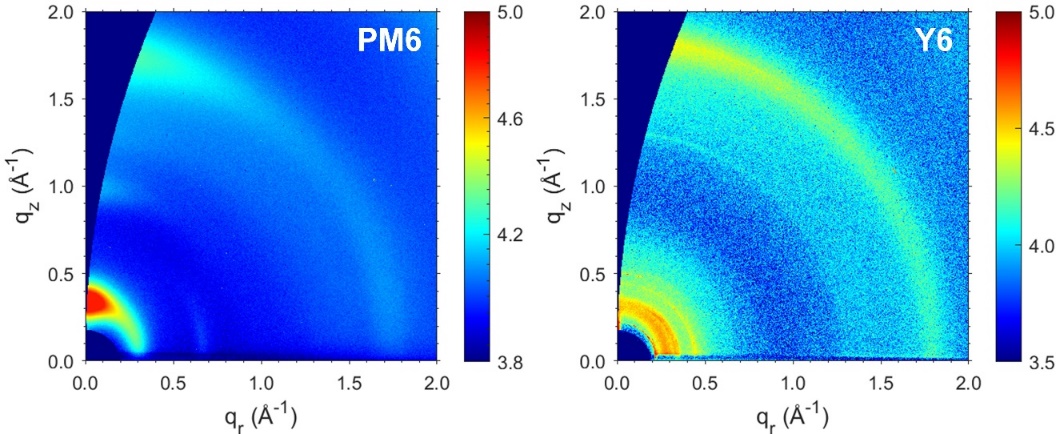


**Figure S5.** 2D Diffraction patterns of the neat PM6 and Y6 films measured by GIWAXS with an incident angle of 0.2º.


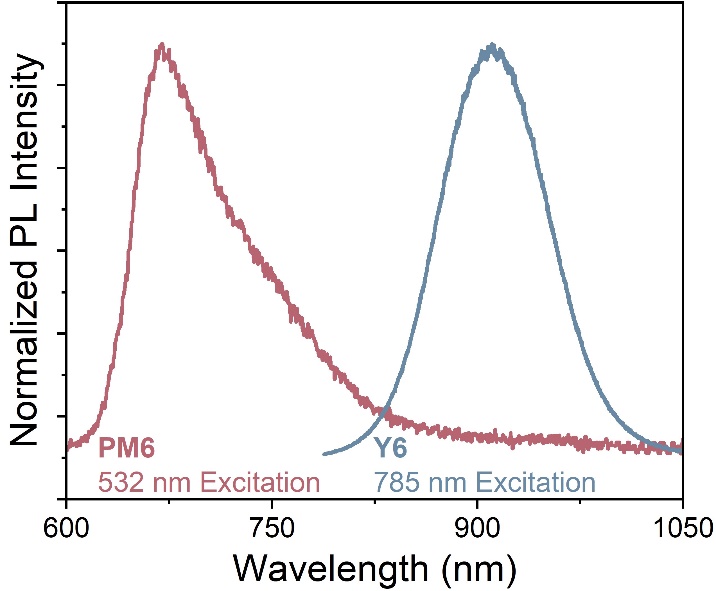


**Figure S6.** PL spectra of neat PM6 and Y6 films excited at 532 and 785 nm, respectively.


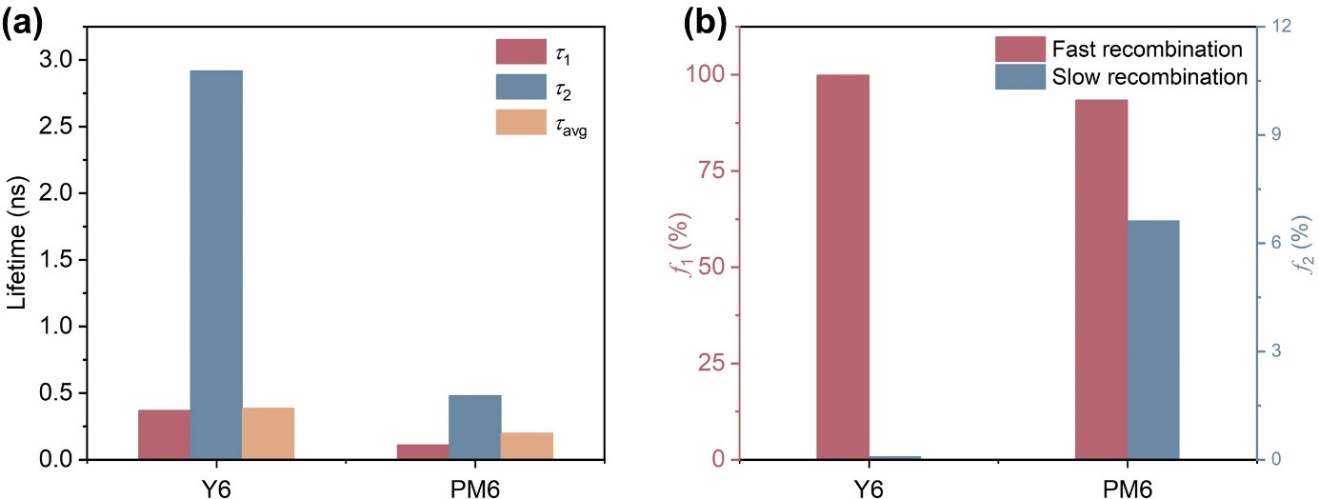


**Figure S7.** TRPL calculations for the neat PM6 and Y6 films. a) Average exciton lifetimes ($\tau_{\mathrm{avg}}$) and the components associated with the fast ($\tau_{1}$) non-radiative recombination/energy transfer and the slow ($\tau_{2}$) radiative/trap-assisted recombination. b) Fractions of each process.


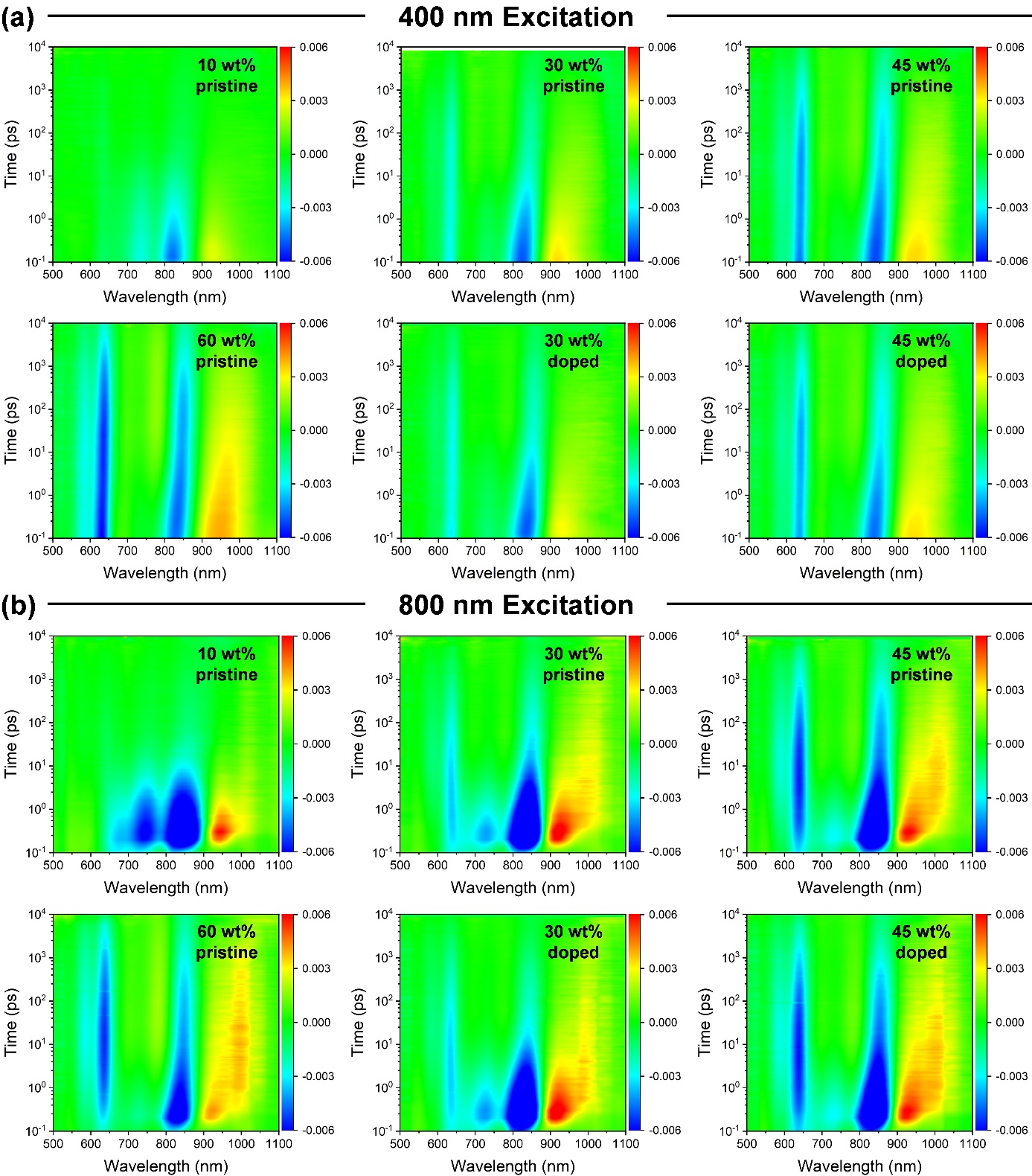


**Figure S8.** 2D TA spectra of the thin films of the pristine and doped PM6(*x* wt.%):Y6 blend films measured at the excitation wavelengths of 400 and 800 nm.


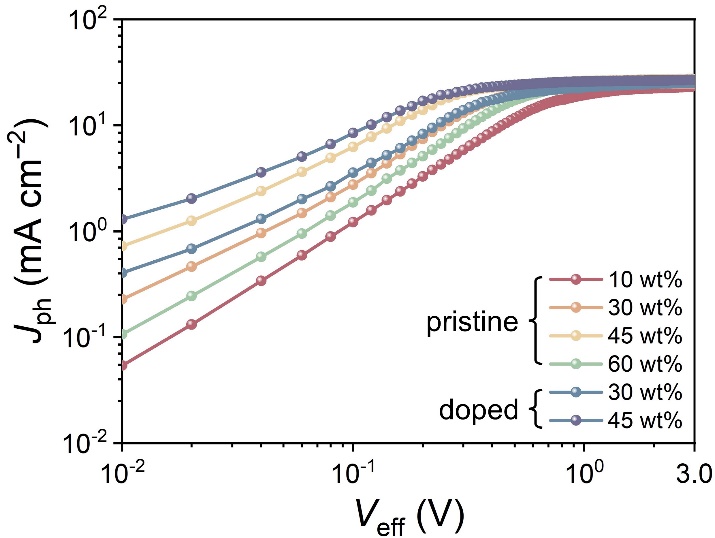


**Figure S9.** *J*_ph_*–V*_eff_ curves of pristine and doped opaque solar cells based on the PM6(*x* wt%):Y6 active layers.


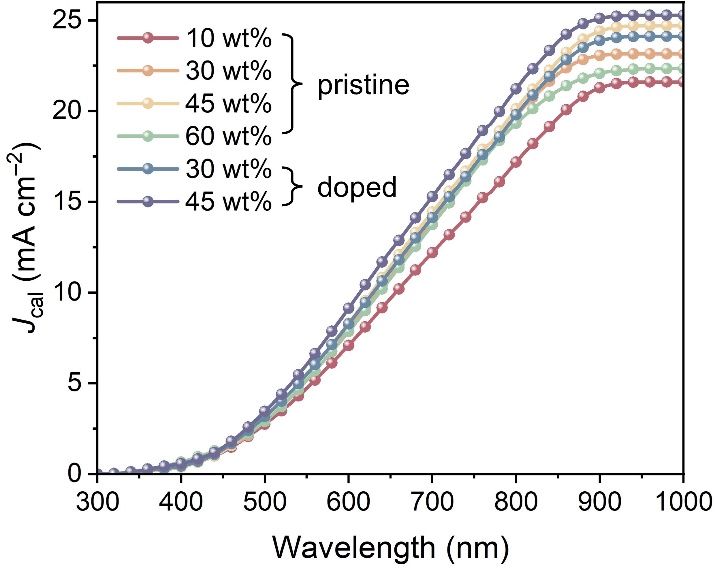


**Figure S10.** Accumulated current density integrated from the EQE spectra of the pristine and doped opaque solar cells based on the PM6(*x* wt%):Y6 active layers.
